# Risks for Acquisition of Bacterial Vaginosis Among Women Who Report Sex with Women: A Cohort Study

**DOI:** 10.1371/journal.pone.0011139

**Published:** 2010-06-15

**Authors:** Jeanne M. Marrazzo, Katherine K. Thomas, Tina L. Fiedler, Kathleen Ringwood, David N. Fredricks

**Affiliations:** 1 Division of Allergy and Infectious Diseases, Department of Medicine, University of Washington, Seattle, Washington, United States of America; 2 Fred Hutchinson Cancer Research Center, Seattle, Washington, United States of America; University of Cape Town, South Africa

## Abstract

**Background:**

Bacterial vaginosis (BV) is common in women who have sex with women. While cross-sectional data support a role for sexual transmission, risks for incident BV have not been prospectively studied in this group.

**Methodology/Principal Findings:**

We studied risks for BV acquisition in a prospective cohort study of women (age 16–35 years) who reported sex with other women (≥1 partner, prior year). Women were followed for one year with examinations at quarterly visits and for genital symptoms at any time. Species-specific 16S rRNA gene PCRs for BV-associated bacteria (BVAB) were applied to vaginal fluid obtained at enrollment. Sexual behaviors were ascertained by computer-assisted interview. Of 335 participants, 239 had no BV at baseline; 199 were seen in follow-up (median follow-up 355 days, 4.0 visits/subject). Forty women experienced ≥1 BV episode. Risks for incident BV were presentation ≤14 days since onset of menses (hazard ratio (HR) 2.3 (95% CI, 1.2–4.7), report of new sex partner with BV history (HR 3.63 (1.1–11.9)), change in vaginal discharge (HR 2.6 (1.3–5.2)) and detection of any of several BVAB in vaginal fluid at enrollment, including BVAB1 (HR 6.3 (1.4–28.1)), BVAB2 (HR 18.2 (6.4–51.8)), BVAB3 (HR 12.6 (2.7–58.4)), *G. vaginalis* (HR 3.9 (1.5–10.4)), *Atopobium vaginae* (HR 4.2 (1.9–9.3)), *Leptotrichia* spp (9.3 (3.0–24.4)), and *Megasphaera*-1 (HR 11.5 (5.0–26.6)). Detection of *Lactobacillus crispatus* at enrollment conferred reduced risk for subsequent BV (HR 0.18 (0.08–0.4)). Detailed analysis of behavioral data suggested a direct dose-response relationship with increasing number of episodes of receptive oral-vulvovaginal sex (HR 1.02 (95% CI, 1.00–1.04).

**Conclusions/Significance:**

Vaginal detection of several BVAB in BV-negative women predicted subsequent BV, suggesting that changes in vaginal microbiota precede BV by weeks or months. BV acquisition was associated with report of new partner with BV; associations with sexual practices – specifically, receptive oral sex – require further investigation.

## Introduction

Bacterial vaginosis (BV) is the most prevalent vaginal infection in reproductive age women, and has been consistently associated with adverse outcomes related to the upper genital tract, and with increased risk of HIV acquisition.[1], [2], [3] Of 3,739 women enrolled during 2001–2004 in a nationally representative sample of the U.S. civilian non-institutionalized population, almost one in three (29.2%; 95% C.I. 27.2–31.3) had BV by Gram stain of vaginal fluid.[4], [5]

Microbiologically, BV is characterized by depletion of hydrogen peroxide-producing lactobacilli that characterize normal vaginal microbiota, with profound overgrowth of anaerobic bacteria.[6] However, the etiology of BV remains elusive. Several cross-sectional studies have reported a wide variety of risks for this common condition, including Black race, douching, smoking, menses, chronic stress, and sexual behaviors (including higher numbers of male sex partners, unprotected vaginal intercourse, anal intercourse, and sex with other women).[7], [8], [9], [10], [11], [12], [13] Fewer studies have followed women prospectively for incident BV. These studies have reported that Black race, report of regular male sex partner or of a female partner during follow-up, vaginal hygiene behaviors, and higher Nugent score at initial evaluation were risks.[14], [15], [16] One study with 619 woman-years of follow-up found that BV acquisition was independently associated with black race, cigarette smoking, vaginal intercourse, receptive anal sex before vaginal intercourse, sex with an uncircumcised male partner, lack of precedent hydrogen peroxide-producing vaginal lactobacilli, and detection of HSV-2 serum antibodies at the visit prior to BV diagnosis.[17]

Women who have sex with women have had a high prevalence of BV (25%–52%) in several cross-sectional studies,[4], [10], [18], [19], [20], [21] We have previously reported risks for prevalent BV in a group of women who reported sex with women in the previous year; these included higher lifetime number of female sex partners, shared use of a vaginally inserted sex toy, and oral-anal sex with a female partner.[10] However, rate of and risks for BV acquisition have not been reported for this group of women. Thus, we assessed risks for BV acquisition in lesbian and bisexual women followed for one year in a prospective study of vaginal microbiota. In addition to measuring the contribution of previously recognized risk factors for BV, we incorporated two novel elements. First, we used comprehensive computer-assisted self-interview (CASI) to assess behavioral risks. CASI has been shown to yield significantly higher rates of disclosure for same-sex behavior and undesirable social behaviors when compared directly with self-administered questionnaires.[22], [23], [24], [25] CASI also provides other benefits, including standardized delivery of survey content, eliminating variation in interviewer or day, and computer-controlled branching, automated consistency and range checking. Second, we applied species-specific qualitative 16S rRNA gene polymerase chain reaction (PCR) assays for various BV-associated bacteria (BVAB)[26] to vaginal fluid obtained at participants' enrollment visit, and considered this as a baseline characteristic in analysis of risks for subsequent acquisition of BV.

## Methods

### Objectives

We aimed to define risks for acquisition of BV among women who were sexually active with other women, with attention to detailed assessment of baseline vaginal microbiota and prospective assessment of behavioral risks, including sexual practices.

### Participants

The study population was composed of women aged 16 to 35 years who reported sex with at least one other woman in the previous year who responded to recruitment through advertisements, media, and community referral between October 2003 and December 2006.

### Investigations Undertaken

At enrollment, subjects completed an extensive computer-assisted self-interview (CASI) on demographics and medical, reproductive and sexual history. The CASI is self-administered with a computer providing text and directly recording respondents' answers without an interviewer's participation. The CASI included detailed information on number and gender of recent and lifetime sex partners, douching practices (history of douching, frequency, indication, solution), frequency of specific sexual practices, menstrual history, hormonal contraception, and antibiotic use. Participants underwent standardized examination including collection of vaginal fluid for Gram stain, saline microscopy, pH measurement, potassium hydroxide evaluation, and culture of *Lactobacillus* species and *Trichomonas vaginalis*. Cultures for *Lactobacillus*, and determination of hydrogen peroxide production, were performed as previously described.[27] To obtain specimens for bacterium-specific PCR assays, a polyurethane foam swab (Catch-All, Epicentre Biotechnologies, Madison, WI) was brushed against the lateral vaginal wall and re-sheathed and frozen immediately in a −80° freezer until DNA extraction. Bacterial vaginosis was diagnosed if three of four clinical (Amsel) criteria (vaginal pH>4.5, clue cells on saline microscopy >20% of epithelial cells, amine odor on addition of potassium hydroxide, and homogeneous vaginal discharge) were present[28] and Gram stain of vaginal fluid confirmed abnormal microbiota (Nugent score >3).[5] Vulvovaginal candidiasis was diagnosed if participants reported a recent change in vaginal discharge or vulvar itching, and if 10% KOH preparation of vaginal fluid demonstrated yeast forms. All participants were asked to return for three subsequent quarterly visits (total duration of follow-up, one year) or for evaluation at any time if genitourinary symptoms occurred. Women with BV at any time during the study were treated with vaginal metronidazole gel (37.5 mg nightly for 5 days) or, if metronidazole was not tolerated, vaginal clindamycin cream (2% (5 g) nightly for 7 days).

#### Microbiology

For DNA extraction, vaginal swabs for bacterial PCR were placed in 15 mL conical vials with 2 mL of saline and vortex mixed for 1 minute to dislodge cells. The solution was centrifuged at 14,000 rpm for 10 minutes, and the pellet resuspended in 100 µl supernatant. DNA was extracted from the pellet using the Ultra Clean Soil DNA Kit (MoBio, Carlsbad, CA) according to manufacturer's instructions. DNA was eluted from silica columns in a volume of 150 uL buffer. Sham digests using a swab without human contact were performed with each round of DNA extraction (every 10–25 samples) to control for contamination that may arise from kit reagents or collection swabs.

Bacterium specific PCR assays were developed based on detection of species-specific regions of the 16S rRNA gene. rDNA sequences from vaginal bacteria detected by broad range rDNA PCR were aligned.[29] Primers were designed to target highly variable regions of the bacterial 16S rRNA gene that appear unique for each species. PCR assays were developed for 17 bacterial species that were commonly detected in vaginal samples.[26] Each 50 µl PCR reaction contained 1X PCR Buffer II, 2 mM magnesium chloride, 0.8 mM deoxyribonucleotide triphosphate mix, 1 unit AmpliTaq Gold DNA polymerase (all from Applied Biosystems, Foster City, CA), 0.2 uM each of forward and reverse primer, and 1 uL of template DNA. PCR conditions included a pre-melt at 95°C for 10 minutes; then 40–45 cycles of 95°C for 30 seconds (melt), 53–62°C for 30 seconds (annealing), and 72°C for 30 seconds (extension); followed by a final extension at 72°C for 7 minutes. PCR products were visualized after electrophoresis in 2% agarose gels and staining with ethidium bromide. Bacterial PCR assays were optimized so that each assay was capable of detecting ≤100 molecules of cloned 16S rDNA per reaction, though most assays could detect 1–10 molecules. Every PCR with a visible band of the expected size on gel electrophoresis was sequenced (BigDye version 3, Applied Biosystems) to confirm that the PCR product had at least 99% similarity with the expected bacterial target, thereby assuring bacterial specificity. PCR reactions without visible bands on gel electrophoresis or without confirmed sequence homology to the expected target were considered negative. No-template PCR controls (consisting of master mix, primers, and water) and sham digest controls (template consisting of water subjected to DNA extraction) were run with each PCR assay to monitor for contamination.

Each subject's extracted DNA was subjected to a human β-globin or 18S rRNA gene PCR to assure that amplifiable DNA was successfully extracted from the sample and to monitor for PCR inhibitors.[30]

Participants were tested at enrollment for *C. trachomatis* and *N. gonorrhoeae* using the APTIMA- COMBO 2 assay (Gen-Probe, San Diego, CA) on urine, and at follow-up visits if they reported interim risk behavior (new sex partner, >1 partner) or genitourinary symptoms.

### Ethics

Written informed consent was obtained from all participants. Conduct of the study adhered to standard guidelines for research involving human subjects, and was approved annually by the University of Washington Human Subjects Review Committee.

### Statistical methods

For the analysis, incident BV was defined by the first visit at which a woman was found to have BV (whether at a quarterly routine visit or at a visit self-initiated for vaginal symptoms) defined by Amsel criteria. After detection of an incident BV episode in a participant, that participant was censored from the analysis. For subanalysis, abnormal vaginal microbiota was defined by Nugent score >3. We used Cox regression analysis to generate hazard ratios for risk of BV acquisition. Adjustment was performed for other statistically significant covariates. All statistical tests were two-sided and a level of p<0.05 was considered statistically significant. Analyses were performed using R 2.4.1 and Stata 9.2 (College Station TX, USA).

## Results

**Figure 1:**
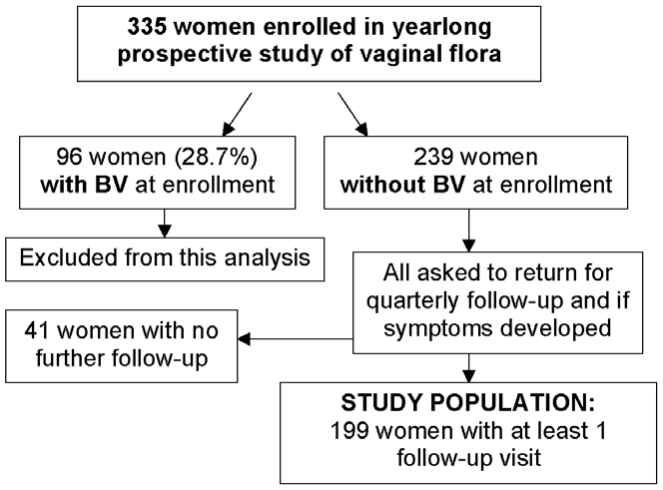
Study flow diagram.

**Figure 2:**
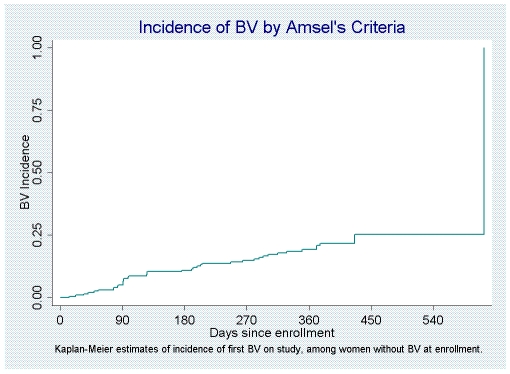
Incidence of bacterial vaginosis (BV) among women with no BV at study entry.

The characteristics of 335 women enrolled in the overall study are summarized in Table 1. Median age was 27 years, 24% self-categorized as non-White, 1/3 reported a prior diagnosis of BV, and 24% reported recent (past 90 days) sex with a male partner. At enrollment, two women (0.6%) had *C. trachomatis*, and 9 (2.7%) had symptomatic vulvovaginal candidiasis. None had *N. gonorrhoeae*, trichomoniasis, or clinically evident genital herpes. Ninety-six women (28.7%) had BV at enrollment and were excluded from the longitudinal analysis of risks for BV acquisition, which thus included 239 participants, as outlined in Figure 1. Of these 239 women who did not have BV at the enrollment visit, 199 returned for at least one follow-up visit. Median duration of follow-up for these 199 women was 381 days (interquartile range, 361–412 days), with a median number of four follow-up visits. Forty episodes of BV occurred among these 199 women over a total of 172.6 woman-years at risk (overall incidence, 0.23 per woman-year), with a median number of days to BV diagnosis after a visit at which BV was not detected of 92 days (interquartile range, 89–104 days). Figure 2 depicts the incidence of BV among participants over the course of follow-up.

**Table 1:** Characteristics of 335 subjects according to presence of bacterial vaginosis at enrollment.

	BV* present	BV absent
	N = 96	N = 239
Age – years		
Median	25	25
Range	15–35	17–34
Race (self-defined) – no. (%)†		
White	69 (72)	185 (77)
Black	7 (7)	9 (4)
Nonwhite, other than black	17 (18)	39 (16)
Declined to provide race data	3 (3)	6 (3)
Sex with women, prior 3 months – no. (%)	80 (84)	197 (82)
Female sex partners, prior 90 days		
Median no.	1	1
Range	0–7	0–5
Sex with men, prior 3 months – no. (%)	19 (20)	61 (26)
Male sex partners, prior 90 days‡		
Median no.	0	0
Range	0–5	0–4
Consistent condom use – no (%)	6 (38)	10 (19)
Current cigarette smoking	33 (40)	66 (32)
Douching, past month	5 (5)	9 (4)
Hormonal contraception use, past 60 days	9 (10)	26 (11)
Vaginal symptoms§ present – no (%)	34 (35)	51 (21)
Concurrent genitourinary infection present – no (%)		
Vulvovaginal candidiasis	2 (2)	18 (8)
Trichomoniasis	0 (0)	0 (0)
* Chlamydia trachomatis*	2 (2)	1 (<1)

*BV  =  bacterial vaginosis at the enrollment visit; defined by presence of Amsel criteria and confirmed by Nugent score >6. The 239 women without BV comprised the sub-population eligible for the prospective analysis of subsequent BV acquisition.

†Subjects were permitted to choose more than one category to describe their race. “White” refers to those who chose only ‘White’ to describe their race; “Black” refers to those who chose “Black or African American” even if they also chose another race, and “Nonwhite” refers to those who chose any other race besides white.

‡Among women who reported vaginal intercourse with a male partner in the prior three months.

§Defined as change in amount, color, or odor of vaginal discharge.

The relationship between participants' characteristic and incident BV in univariate analysis is shown in Table S1. Detection of any of several BVAB at the enrollment visit, when participants were free of BV, was associated with the highest risks of subsequent BV; these included BVAB1, BVAB2, BVAB3, *G. vaginalis*, *A. vaginae*, *Leptotrichia* spp, and *Megasphaera*-1. Detection of *Lactobacillus crispatus* by PCR assay or of H_2_O_2_-producing lactobacilli by cultivation at enrollment was associated with reduced risk. Other significant risks for incident BV included examination in the two weeks following menses, report of a new female sex partner with a history of BV, and report of recent change in vaginal discharge. Despite the latter finding, most incident BV occurred in asymptomatic women (28 of 40 cases (70%)).

Detailed analysis of sexual behavioral data revealed a direct dose-response relationship with increasing numbers of reported episodes of receptive oral-vulvovaginal sex. The corresponding estimated relative risk (RR) of 1.02 per act translates to a 21% increase in risk of BV for each additional 10 such acts in the last three months. A similar association with receptive oral-anal sex did not reach statistical significance (relatively few women reported this behavior). Additional behaviors assessed for which no association with BV acquisition was seen included other sexual activities noted, and time to last episode of each of the behaviors (in addition to sharing sex toys, shown) in Table 1. Of note, the median time to reported last episode of the specific sexual behaviors listed in Table 1 ranged from 4 days (any sex) to 21 days (receptive anal sex).

Detection by PCR assay at enrollment of any of the BV-associated bacteria listed in Table 1 was highly correlated with the likelihood of other positive BV-associated bacteria PCR assays. Spearman correlation coefficients between specific BVAB ranged from 0.5 to 0.77, with many greater than 3.0. Thus, the multivariate model of risks for acquisition of BV included a composite variable that was based on detection of any positive PCR assay for the bacteria that were significantly associated with subsequent BV (as listed in Table S1). Table 2 summarizes the results of the multivariate analysis (Table 2). In an analysis that adjusted for report of new sex partner with a history of BV, detection of any of BVAB1, BVAB2, BVAB3, *P. lacrimalis*, *Leptotrichia* species, *Atopobium* species, *G. vaginalis Megasphaera*-1, and *L. crispatus* remained significantly associated with BV acquisition.

**Table 2:** Multivariate analysis of risks for acquisition of bacterial vaginosis (BV).

Characteristic	Hazard ratio	P-value
	(95% CI)	
Enrollment PCR+ for BVAB1, BVAB2, BVAB3, *P. lacrimalis*, *Leptotrichia*, *Atopobium*, *Megasphaera* spp or *G. vaginalis*	3.02 (1.12–8.13)	0.03
Enrollment PCR+ for *L. crispatus*	0.16 (0.07–0.36)	<0.001
≤14 days since start of menstrual cycle	3.49 (1.36–8.93)	0.01
New sex partner with history of BV	2.96 (0.38–23.2)	0.30

## Discussion

Among women without BV who were subsequently followed for an average of one year, we found that detection of several BV-associated bacteria (BVAB) in vaginal fluid at the enrollment (BV-negative) visit predicted subsequent acquisition of BV. Conversely, detection of *L. crispatus* at enrollment was associated with reduced risk of subsequent BV acquisition. Women were also more likely to have BV diagnosed when they presented in the two weeks after menses. The significant association between detection of BVAB at enrollment and BV acquisition remained significant after adjusting for timing of examination relative to participants' menstrual cycles and new sex partner with a history of BV. Notably, although over 80% of participants without BV at enrollment had *Lactobacillus crispatus* at enrollment as detected by PCR assay, 20% acquired BV in the year of follow-up. The incidence of BV that we observed in our participants—0.23 episodes/woman-year—was only slightly lower than that reported in a larger yearlong prospective study of young heterosexual women.[17]

Despite collection of extensive sexual risk behaviors using CASI during follow-up, only report of a new female sex partner who provided a history of BV was clearly associated with increased risk of BV acquisition in univariate analysis. The dose-response relationship we observed between risk of BV acquisition and increasing reported number of episodes of receptive vulvovaginal sex is of interest (as is the less significant but directionally similar association with receptive oral-anal sex), although we did not observe these associations when these behaviors were analyzed using other measures (for example, report of any behavior vs. not, or time to last behavior). Thus, these findings support further detailed investigation of the potential role of these behaviors in future BV acquisition studies, to confirm the associations we detected. Given the absence of associations between other recent sexual practices and BV acquisition, the significance of association between BV and report of a new female partner with BV history remains unclear. As we have previously reported, an intervention to prevent sexual transmission of vaginal fluid in this cohort – despite succeeding in reducing the risk of sexual practices targeted by the intervention—failed to reduce the risk of BV acquisition.[31] Our inability to establish a role for sexual transmission of BV in this cohort may relate to several factors, including imprecise assessment of the time of sexual exposure to acquisition of a “precipitant” for BV, the possibility that multiple sexual practices may be involved and are often practiced concurrently, or, as is highly likely, that BV pathogenesis is multifactorial: while unprotected sex is likely contributory, other factors are probably involved.

We have previously reported that among women with BV in this cohort who were treated with vaginal metronidazole, predictors of treatment failure included detection of specific BVAB at baseline, including the Clostridia-like bacteria BVAB1, BVAB2 and BVAB3, *Peptoniphilus lacrimalis* and *Megasphaera* phylotype 2, as well as failure to adhere to five days of vaginal metronidazole.[32] Importantly, report of no specific sexual practices with either male or female partners in the month after treatment predicted either persistent bacterial vaginosis or abnormal microbiota. The latter finding is notable, as others have reported that unprotected vaginal intercourse is associated with recurrent BV. However, as we note below, our ability to detect such associations was limited by the relatively small number of outcomes we observed and the selected population we studied.

Our observation that detection of specific BVAB in vaginal fluid weeks to months prior to a diagnosis of BV suggests that changes in vaginal microbiota may precede the development of BV by a considerable time period. Bacterial vaginosis is a heterogeneous syndrome characterized by diverse microbiota, some of which might be essential “founder” species necessary to establish an environment favorable to progression to BV. For example, a tenacious biofilm on vaginal epithelium may lead to antibiotic failure or create a persistent reservoir of bacteria further increasing women's risk of treatment failure.[33] Concentrations of bacteria at the earliest stages of biofilm formation may be insufficient to cause BV, yet may provide a critical layer of bacterial cells that facilitate attachment of other members of the bacterial community. These early colonizers may be detected by species-specific PCR assays as described above. In some women assessed frequently (for example, daily), rapid shifts in vaginal microbiota as measured by quantitative PCR assays and by Nugent score are evident.[34] Studies focused on the kinetics of acquisition of vaginal bacterial communities are likely to be very useful for understanding the pathogenesis of BV.

In our participants, BV was not associated with risk factors that others have identified, including failure to use condoms[14], [16], [35], [36] and other sexual behaviors with male partners.[17] This may partly be explained by a low incidence of sexual contact with men among our subjects in the month after treatment. However, given the high prevalence of BV among lesbians, the frequency of concordant BV in female sex partners, and the observation that report of sex with another woman was associated with increased risk of recurrence in one prospective study,[14] we were surprised not to find a clearer association between BV acquisition and sexual behaviors with female partners in the month after treatment. This could be explained by small numbers of women in some subgroups, or by an overriding role of baseline vaginal microbiota associated with BV promoting persistence in this group —rather than “reinfection” or exposure to another causative factor through sex[14], [16], [35], [36].

### Limitations

The main limitation of our analysis is that PCR assays for individual BVAB in vaginal fluid were obtained only at the enrollment visit; it is likely that more proximate detection of these bacteria in women without BV to the actual BV episode will demonstrate an even higher risk for subsequent BV acquisition. Prospective studies that apply PCR assays to frequent serial measurements—ideally through self-collection of vaginal swabs—would be the ideal approach to clarifying the temporal sequence of events that lead to BV. Nonetheless, several of the BVAB we detected at the enrollment visit were associated with markedly elevated risk of subsequent BV. Second, the interpretation of our multivariate analysis is limited by two factors: the relatively small numbers of women who had some individual BVAB detected by PCR assay, and the high degree of correlation among BVAB in that each species is frequently found as part of a larger community of BVAB. For this reason, we did not analyze individual BVAB in our multivariate analysis. Third, our subjects were selected on the basis of reporting sex with other women. Although 24% also reported sex with men in the three months prior to enrollment, they are unlikely to be representative of exclusively heterosexual women, a group that should be studied in similar fashion and to whom our findings may not necessarily apply. However, the fact that our subjects infrequently reported vaginal intercourse with male partners in the month after treatment might actually be advantageous in helping to define the role of vaginal microbiology in determining whether antecedent vaginal colonization of these bacteria have an independent role in predicting future incidence of BV. Finally, we relied on self-report of sexual behaviors to estimate subsequent BV risk, although we used CASI to minimize under- or over-reporting of these behaviors.

Our findings raise several important areas for future research. First, comparative assessments of risks for acquisition of BV, including molecular characterization of baseline microbiology of BVAB, need to be performed in heterosexual women; these efforts should help to define whether the molecular epidemiology of bacterial vaginosis differs from that of women who have sex with women, and to further help define a potential role of unprotected vaginal intercourse with male partners in BV incidence. Second, our BVAB-specific assays were qualitative. Quantitative polymerase chain reaction assays applied to vaginal fluid samples obtained in our subjects may offer more precise predictive value for incident BV, though they are typically less specific than performing sequence analysis on the amplicons as was performed in this study. Third, more frequent sampling (for example, daily or weekly) for application of bacterium-specific PCR assays in women without BV should help to better define the evolution of vaginal microbiota towards the “tipping point” of actual BV; these measures should be thoughtfully integrated with concomitant collection of behavioral data to further explore potential links with sexual practices. Finally, the establishment of consensus definitions for incidence, persistence and recurrence of BV would assist in progress towards describing the natural history of this condition. Whether risk factors that promote incidence are the same as those that promote persistence or recurrence is not clear.

In summary, our findings suggest that vaginal colonization with key BVABs among women with no BV is an independent risk factor for incident BV. Because BV may confer an increased risk of poor pregnancy outcome and HIV acquisition and predict upper genital tract disease, defining microbiological risk factors for new episodes of BV may offer new approaches to prevention.

## Supporting Information

Table S1 Associations between participants' characteristics & acquisition of bacterial vaginosis (BV) in univariate analysis.
(0.0 B DOC)
