# Supplementary material for: Risks for Acquisition of Bacterial Vaginosis Among Women Who Report Sex with Women: A Cohort Study
Source: PLoS One. 2010 Jun 15;5(6):e11139. doi: 10.1371/journal.pone.0011139 (PMC2886123; doi:10.1371/journal.pone.0011139)
Supplement: Table S1 — Associations between participants' characteristics & acquisition of bacterial vaginosis (BV) in univariate analysis. (0.0 B DOC) [file pone.0011139.s001.doc]

Table S1. Associations between participants’ characteristics & acquisition of bacterial vaginosis (BV) in univariate analysis

| **Characteristic** | **Prevalence^*^ (%)** | **BV incidence^** | **Hazard Ratio (95% CI)** | **P-value** |
| --- | --- | --- | --- | --- |
| Enrollment | | | | |
| **ALL PARTICIPANTS** |  | 0.23 |  |  |
| **Black race** | 3% | 0.22 | 1.0 (0.14-7.34) | >0.99 |
| **Enrollment PCR assay^#^ + for:** |  |  |  |  |
| **BVAB1** | 4 (3) | 1.24 | 6.3 (1.4-28.1) | 0.02 |
| **BVAB2** | 8 (6) | 2.35 | 18.2 (6.4-51.8) | <0.001 |
| **BVAB3** | 3 (2) | 2.27 | 12.6 (2.7-58.4) | 0.001 |
| **P. lacrimalis** | 20 (16) | 0.48 | 2.7 (1.1-6.4) | 0.03 |
| **G. vaginalis** | 70 (55) | 0.37 | 3.9 (1.5-10.4) | 0.007 |
| **Atopobium spp** | 26 (20) | 0.67 | 4.2 (1.9-9.3) | <0.001 |
| **Leptotrichia spp** | 10 (8) | 1.46 | 9.3 (3.6-24.4) | <0.001 |
| **Megasphaera-1** | 12 (9) | 1.64 | 11.5 (5.0-26.6) | <0.001 |
| **L. iners** | 101 (80) | 0.28 | 3.6 (0.8-15.2) | 0.08 |
| **L. crispatus** | 104 (82) | 0.15 | 0.18 (0.08-0.4) | <0.001 |

| **Characteristic** | **Prevalence^*^ (%)** | **BV incidence^** | **Hazard Ratio (95% CI)** | **P-value** |
| --- | --- | --- | --- | --- |
| Enrollment | | | | |
| **Growth of *Lactobacillus* species in culture** | | | | |
| **Any lactobacilli** | 178 (95) | 0.23 | 0.99 (0.2-4.1) | >0.9 |
| **Hydrogen peroxide-producing** | 162 (86) | 0.19 | 0.3 (0.15-0.61) | 0.001 |
| **Quantity of lactobacilli, log10, median** | 7.9 |  | 0.99 (0.85-1.16) | 0.94 |
| **Quantity of hydrogen peroxide-producing lactobacilli, log 10, median** | 7.0 |  | 0.84 (0.7700.93) | 0.001 |

| **Characteristic** | **Prevalence* (%)** | **BV incidence^** | **Hazard Ratio**  **(95% CI)** | **P-value** |
| --- | --- | --- | --- | --- |
| **Since Previous Visit** | | | | |
| **Current smoker** | 41 | 0.26 | 0.89 (0.47-1.68) | 0.72 |
| **<14 days since start of menstrual cycle @ exam** | 50 | 0.32 | 2.32 (1.16-4.68) | 0.02 |
| **Change in vaginal discharge** | 16 | 0.51 | 2.56 (1.27-5.17) | 0.009 |
| **New female partner with history of BV** | 2 | 0.94 | 3.63 (1.11-11.9) | 0.03 |
| **Same female partner with history of BV** | 3 | 0.51 | 2.55 (0.61-4.68) | 0.20 |
| **Vaginal lubricant use** | 58 | 0.26 | 1.06 (0.50-2.26) | 0.88 |
| **Sharing sex toys** | 17 | 0.25 | 1.15 (0.51-2.63) | 0.74 |
| **< 30 days since last receptive use** | 11 | 0.41 | 2.17 (0.94-4.97) | 0.07 |

| **Characteristic** | **Prevalence* (%)** | **BV incidence^** | **Hazard Ratio (95% CI)** | **P-value** |
| --- | --- | --- | --- | --- |
| **Since Previous Visit** | | | | |
| **Receptive oral sex** | 66 | 0.23 | 0.88 (0.46-1.69) | 0.70 |
| **Dose response, per act** | -- | -- | 1.02 (1.00-1.04) | 0.05 |
| **Any receptive anal sex** | 29 | 0.18 | 0.73 (0.35-1.55) | 0.42 |
| **Any oral-anal sex** | 16 | 0.21 | 0.75 (0.29-1.92) | 0.55 |
| **Dose response, per act** | -- | -- | 1.05 (1.00-1.10) | 0.06 |
| **Digital-vaginal sex** | 81 | 0.23 | 0.74 (0.35-1.58) | 0.45 |
| **Vaginal intercourse (male)** | 23 | 0.21 | 0.79 (0.36-1.72) | 0.55 |
| **Without condom** | 18 | 0.19 | 0.67 (0.26-1.71) | 0.40 |

* “Prevalence” for characteristics in “since previous visit” category refers to percent of time participants reported the given risk characteristic. Additional behaviors assessed for which no association with BV acquisition was seen included: any sexual activity and time to last episode of each of the behaviors (in addition to sharing sex toys, shown) in Table

^ Incidence represents episodes per woman-years at risk for BV

# Number of women testing positive is shown for each PCR assay, as 127 of all 199 women (64%) were tested for BVAB by PCR.
